# U.S. and Swedish primary care physicians’ views on promoting healthy lifestyles: a qualitative study

**DOI:** 10.1186/s12875-026-03407-1

**Published:** 2026-06-17

**Authors:** Helene Johansson, Lars Weinehall, James Dalton, Julie Sorensen, Paul Jenkins, Lars Jerdén

**Affiliations:** 1 Department of Epidemiology and Global Health, Umeå University, Umeå, 901 87 Sweden; 2 Bassett Research Institute, Mary Imogene Bassett Hospital, Cooperstown, NY 13326 USA; 3 Department of Health and Welfare, Dalarna University, Falun, Sweden

**Keywords:** Counseling, Lifestyle, Prevention, Health promotion, Primary care, Smoking, Alcohol, Qualitative methods, Sweden, USA

## Abstract

**Background:**

Primary care patients can benefit from counseling to address unhealthy lifestyle habits. Despite this, evaluations most often note that primary care facilities are not using the full potential of healthy lifestyle counseling methods. Studies comparing prevention efforts in different countries generally find substantial differences between countries. Quantitative studies carried out in a project targeting health professionals and patients in the U.S. and Sweden have demonstrated considerable differences between the two countries. In particular, U.S. health care professionals were found to be more engaged in lifestyle counseling than their Swedish counterparts. The aim of this qualitative study was to more fully explore and compare U.S. and Swedish primary care physician’s views on and involvement in promoting healthy lifestyles.

**Methods:**

In total, 28 tape-recorded, semi-structured interviews were carried out with Swedish and U.S. primary care physicians. Data were analyzed using a combination of inductive and deductive qualitative content analysis approaches.

**Results:**

Primary care physicians in both countries viewed addressing lifestyle habits like smoking, lack of physical activity and poor diet as important, but their level of commitment ranged “from dedicated to merely doing one’s duty”. This overarching theme was reflected across categories concerning role responsibility, client receptiveness and perceived impact, self-efficacy, supporting mechanisms, and working conditions. U.S. physicians were generally more engaged in promoting healthy lifestyles and described more structured routines and greater personal responsibility, whereas Swedish physicians more often relied on referral options and shared responsibility across professional groups. In both countries, lifestyle change was seen as difficult, and physicians’ involvement was shaped by confidence, skills, time, and available support. Limited resources and constrained working conditions, particularly lack of time, were barriers contributing to doubts about the effectiveness of counseling efforts.

**Conclusions:**

There was consensus in both countries about addressing lifestyle habits as an integrated part of their role as primary care physicians. The study suggests that physicians were uncertain about the effectiveness of their interventions concerning unhealthy lifestyles, implying a need for further dissemination of the latest evidence for such interventions. Physicians requested a team approach for promoting healthy lifestyles with a clearly defined role for physicians in these efforts.

**Supplementary Information:**

The online version contains supplementary material available at 10.1186/s12875-026-03407-1.

## Background

Lifestyle habits can be defined as everyday behaviors that an individual can influence. Unhealthy lifestyle habits, such as smoking and poor eating habits, have a major impact on the burden of disease [1]. Family practice settings can utilize effective methods, mainly counseling, to address unhealthy lifestyle factors, which have been available since the 1970s [2]. There has been increasing research in this field more recently, and there are now effective methods for primary care treatment of key unhealthy lifestyle habits (smoking, harmful alcohol consumption, unhealthy eating habits and insufficient physical activity) [3, 4].

The role of primary care in the promotion of healthy lifestyles has been evaluated and discussed for many years [5–9]. Despite the increasing interest in this area, evaluations indicate that primary care practices aren’t using the full potential of these proven methods, i.e. too few patients get access to counseling in primary healthcare settings [8, 10–16].

There has also been an interest in comparing prevention efforts in different countries, in order to formulate hypotheses for research and quality improvement [5, 6, 8, 10, 13]. Such studies generally find substantial differences between countries, which can provide helpful information on their effectiveness. Thus, a Swedish–U.S. comparison was chosen for its potential to improve the medical community’s understanding of how to provide this support and possible outcomes. The research has been carried out in primary care settings in Sweden, and at the Bassett Healthcare Network (BHN), a healthcare delivery system in New York State in the United States. Both settings represent health systems in high-income countries with well-developed primary care programs, but they differ substantially in how prevention and lifestyle counseling are institutionally framed within clinical practice.

In this project, attitudes and practices in primary care settings were compared [17, 18]. A survey of health professionals demonstrated large differences between the two countries, with U.S. professionals being more engaged in counseling on lifestyle habits. U.S. physicians rated the importance of counseling higher, as well as the expertise they possessed in providing this support [17]. Telephone interviews with patients showed a similar picture, as the proportion of U.S. patients reporting their primary care provider had initiated a discussion of lifestyle habits was, except for alcohol, roughly double the level reported by the Swedish patients [18].

Thus, there is a need to expand the promotion of healthy lifestyle habits in primary care. To that end, comparisons between countries can help in formulating hypotheses or offering evidence for methods of improvement. Our research project [17, 18] has raised questions concerning physicians´ views and practices in addressing unhealthy lifestyles and demonstrated differences between Sweden and the U.S. in this respect. The aim of this qualitative interview study was to more fully explore and compare U.S. and Swedish primary care physician’s (PCP´s) views on, and involvement in promoting healthy lifestyles. Based on our knowledge of the literature, this is the first qualitative study to compare physicians’ thoughts on their role in supporting healthy lifestyles in primary care settings in different countries.

## Methods

The study utilized a qualitative research design, gathering data in semi-structured interviews with Swedish and U.S. PCP´s. The moderator’s guide included a case study vignette (i.e., a written description of patient visit scenarios), which offered the possibility to explore and compare physician’s perceptions and beliefs about providing healthy lifestyle counseling to patients [19].

### Study settings

The study was carried out in primary care settings in the counties of Dalarna and Stockholm (Sweden), and in the eight county region covered by the BHN in New York State (United States). In Sweden, the county councils are responsible for the delivery of healthcare to county residents. Healthcare is financed primarily by taxes and delivered either by the county councils’ own units or by providers with which the county council has made agreements. The provider receives most of its income through capitation, and a smaller amount from visit-based payment. Even if the two counties are different in terms of geographical location and employment structure, according to official statistics they together reflect conditions that are fairly representative for Sweden (e.g. education, economic conditions, lifestyle and morbidity). BHN is an integrated healthcare delivery system that provides primary and comprehensive services to a large group of patients in Central New York State.

At the BHN, there are currently no financial incentives to either the physicians and mid-levels or their health clinics for providing counseling on disease prevention. In the Swedish system, the professional does not receive financial incentives for providing counseling, however, their health center typically receives a small incentive. Physicians in both settings are salaried employees within integrated health systems, rather than working under fee-for-service reimbursement. This makes it possible to compare physicians who operate under relatively similar employment arrangements, but within different health system contexts, thereby illuminating how institutional and cultural settings shape physicians’ views on the management of unhealthy lifestyles.

### Recruitment of informants

Physician recruitment sought to provide maximum variation in sex, age, clinical experience and employment status, as well as health center environments. In Sweden, the selection of potential participants was facilitated by author LJ, who had been working for many years as a family physician for the County Council of Dalarna. In the U.S., the selection of potential participants was done by an experienced administrator at the Bassett Research Institute. Potential participants were contacted by email, although some of them were also contacted by phone. Oral and written information about a study of physicians´ communication with patients was provided to participants, and a majority of invited physicians agreed to take part in the study. No incentives for participation were provided. In Sweden, physicians from seven clinics (both private and those run by the County Council), were interviewed. In Sweden, permanent staff, residents and non-residents took part in interviews. In the U.S., physicians from six clinics in the Bassett Healthcare Network were interviewed. One clinic was situated at the hospital in Cooperstown, while the others were located in rural communities. Recruitment ended when saturation was achieved in both countries, i.e. when interviews did not generate substantially new information about the topic. Participants´ characteristics are shown in (Table 1).

**Table 1:** Characteristics of the physicians interviewed

Sex	Age	Country of birth	Country medical education	Specialties	Country and service provider*	Work %	Clinical %	Employment status
M	62	Sweden	Sweden	Family Medicine	Sweden (private)	100	100	Permanent
M	44	Poland	Poland	Family Medicine	Sweden	100	90	Permanent
F	39	Sweden	Sweden	Family Medicine	Sweden (private)	100	100	Permanent
F	44	USA	USA	Internal Medicine	USA	62	62	Permanent
M	60	USA	USA	Internal Medicine	USA	100	100	Permanent
M	62	USA	USA	Internal Medicine	USA	100	60	Permanent
M	42	Sweden	Sweden	Family Medicine	Sweden (private)	100	100	Permanent
F	26	Sweden	Sweden	-	Sweden	100	100	Non-permanent
M	32	Columbia	Lithuania	-	Sweden	100	100	Resident (ST)
F	37	Sweden	Denmark	-	Sweden	100	100	Resident (ST)
M	53	Sweden	Sweden	Family Medicine, Pediatrics	Sweden	100	100	Non-permanent
M	38	Sweden	Sweden	Family Medicine	Sweden	89.5	89.5	Permanent
F	69	USA	USA	Internal medicine	USA	< 50	< 50	Permanent
M	61	USA	USA	Internal Medicine	USA	100	90	Permanent
F	34	USA	USA	Family Medicine	USA	100	90	Permanent
M	59	USA	USA	Internal Medicine, Pediatrics	USA	100	90	Permanent
M	58	USA	USA	Internal Medicine, Geriatrics	USA	90	45	Permanent
F	54	Brazil	USA	Family Medicine	USA	65	65	Permanent
M	38	USA	USA	Internal Medicine, Pediatrics	USA	100	100	Permanent
M	71	USA	USA	Internal Medicine, Geriatrics, Hematology	USA	60	60	Permanent
F	39	USA	Grenada	Family Medicine	USA	100	100	Permanent
F	35	USA	USA	Family Medicine	USA	100	90	Permanent
M	62	USA	USA	Family Medicine	USA	100	100	Permanent
F	53	USA	USA	Family Medicine	USA	60	60	Permanent
M	57	USA	USA	Family Medicine	USA	90	90	Permanent
F	50	USA	USA	Doctor of Osteopathy, Internal Medicine	USA	100	100	Permanent
M	58	Sweden	Sweden	Family Medicine	Sweden	85	85	Permanent
M	52	Sweden	Sweden	Family Medicine	Sweden	100	100	Permanent

* In the U.S., the the Bassett Healthcare Network was the sole service provider. In Sweden, both private service and County Council centers were service providers

### Data collection

In total, 28 tape-recorded interviews were carried out during 2014 and 2015 in the U.S. and Sweden by primary care physician researchers in each setting (LJ and JD). None of the participants were asked to do a second interview. The interviews lasted between 30 and 74 min and took place in clinic rooms that offered privacy. While LJ and JD conducted three interviews together, all other interviews included the informant and the interviewer exclusively. LJ studied qualitative research methodology as part of his research training and had previous experience conducting research interviews. JD received formal training in communication skills and had many years of experiencing lecturing medical residents on how to conduct patient interviews. Interviewers followed a moderator’s guide, although this guide was not pilot tested. The guide consisted originally of two case vignettes and four additional questions. However, since the first case elicited rich data, the second case vignette was eventually excluded.

The interviews started with the presentation of a case study vignette describing a fictitious case: a middle-aged, obese man, previously healthy, whose self-administered blood pressure readings were high [see Additional file 1]. The interviewees were instructed to describe in detail how they would handle such a case, questions they would ask, possible clinical examinations, laboratory tests, diagnoses, referrals, follow-up etc. If the interviewee asked for additional information, (e.g., smoking or physical activity), information about these fictitious habits were revealed by the interviewer. Otherwise, this information was not offered. In addition to the case vignette, four questions were asked at the end of the interview to capture physicians’ views on the importance of and their capacity to discuss lifestyle issues with patients [see Additional file 2]. Field notes were not taken during or after the interviews.

### Data analysis

The audio recordings were transcribed verbatim, in Swedish and English, respectively, with the average word count consisting of 6,300 words. One of the recordings was deleted by mistake. The only written information from this interview was a short summary documented by LJ after the interview. The transcripts were not returned to participants for comments. The transcriptions were reviewed several times to become familiar with the content.

The analysis was performed using a combination of conventional and directed qualitative content analysis approaches [20]. The conventional (inductive) approach was used separately for U.S. and Swedish data sets. The analytical work was carried out in Open Code 4.0, a tool for qualitative data coding, developed by Umeå University. The first step was to identify, condense and interpret relevant meaning units, i.e. words or sentences containing aspects related to each other through their content and context, which were relevant to the research question [21]. These condensed meaning units captured both the descriptive, explicit meaning of the text (manifest content), as well as the underlying, implicit meaning (latent content) [21]. Condensed meaning units with similar content were then sorted into sub-categories and categories. Finally, the categories from both datasets were merged, re-labeled, and organized in a theoretical framework adapted from Laws et al. [22]. The framework suggests that clinicians’ risk factor management practices are shaped by their beliefs about whether they should and can discuss lifestyle issues with patients. These beliefs in turn are influenced by a combination of patient-, provider- and context-level factors. The framework emphasizes a synergetic relationship between two core domains: commitment and capacity. Commitment reflects the priority clinicians place on risk factor management, which are influenced by three key components: perceived role responsibility congruence, patient receptiveness, and beliefs about the likely impact of intervening. Capacity in contrast, is shaped by clinicians’ self-efficacy, available supporting mechanisms and their work environment, and it reflects their perceived ability to address lifestyle issues in practice.

The initial part of analysis was performed by the first author (HJ) and regularly discussed and negotiated with LJ, who carried out most of the interviews; both are fluent in Swedish and English and experienced qualitative researchers with formal training in qualitative methods.

In the latter stages of the analysis process, parts of the Swedish dataset – such as meanings units, quotes and categories – were translated into English, and the findings were then discussed and jointly negotiated by the entire research team. The participants were not asked to review the research findings. However, the findings were presented and discussed at seminars in Cooperstown for physicians of the BHN, and in Sweden at national forums for family physicians.

### Ethical considerations

The U.S. study was approved by the Bassett Healthcare Network Institutional Review Board 2013, March 21. Ethical approval for the Swedish research was obtained from the Regional Ethical Committee at Umeå University (Dnr. 2011-64-31 M). In Sweden, all interviewees signed a written consent form at the beginning of the interview, while in the U.S oral informed consent was obtained, as written consent was not required.

## Results

The data analysis showed that the range and variation in perceptions was greater among individuals irrespective of home country, as compared to differences that could be assigned to physicians in either one country or the other. However, discussions also indicated different views that could be attributed to the health systems (resources, supporting structures, etc.) in the two countries, respectively.

The analysis resulted in one overarching theme with seven associated categories and four subcategories, which were organized into a structure consisting of five key components, adapted from the theoretical framework by Laws et al. [22]. The main findings are presented in Table 2. The Results section follows these headings, and direct quotations from the interviews are included to demonstrate how the interpretation is grounded in the data.

**Table 2:** A description of the theme (bold) with associated categories (normal) and sub-categories (italic), organized into a structure consisting of five key components* adapted from a theoretical framework by Laws et al. [22]

A range from “being dedicated” to merely “doing one´s duty”				
Role responsibility*	Client receptiveness & likely impact of intervening*	Self-efficacy*	Supporting mechanisms*	Working conditions*
Addressing lifestyle - an integrated part of the medical role *Beyond screening and brief advice* *Setting limits on the role of promoting healthy lifestyles* Sharing responsibility across professional groups	Lifestyle habits are hard to change *Confidence in the potential* *Doubts about impact*	Differences in self-efficacy influence counseling	Referral opportunities to specialist services A system in place to routinely address behavioral risk factors	Preventive ambitions constrained by working conditions

### A range from “being dedicated to merely doing one´s duty”

All informants expressed the importance of addressing lifestyle habits, although their expressed level of commitment ranged from “being dedicated” to merely “do[ing] one´s duty”. The level of commitment was to varying degrees related to views that can be attributed to physician’s responsibility, client receptiveness and the likely impact of intervening, self-efficacy, support mechanisms and working conditions.

Physicians who were dedicated to lifestyle counseling were in the minority; however, these individuals strived for preventive care for all patients. They stressed the importance of addressing lifestyle habits even if they were behind on their patient schedule.

*“I´m very passionate about it because preventive care is care. …Preventive care is what I strive for for my patients …” U.S.*

*“I try to incorporate it in daily practice. … I do it even when I’m rushed because I do think it’s important. I even do it when I’m behind*,* and that’s probably why I’m behind all the time. Because I think it’s really important.” U.S.*

*“That’s what I´m doing all the time*,* almost every patient.” Sweden*.

Those physicians who were simply “doing one’s duty” also believed that preventive care was important, but often preferred focusing on the treatment of diseases, addressing lifestyle habits only when patient’s shared concerns or if time permitted.

*“I will not inquire enough about behaviours if the patient has several medically active concerns.” U.S.*

*“Hopefully*,* I ask about lifestyle if it´s related to the illness*,* otherwise I skip it.” Sweden*.

A few informants wished to increase the time available for lifestyle habits while at the same time realizing that this is probably not realistic. However, for most of them, it was not a question of spending more time on it but doing it better. Accordingly, they asked for better training and support systems.

### Role responsibility

#### Addressing lifestyle - an integrated part of the medical role

All informants considered addressing lifestyle habits as an integrated part of their medical role, especially pointing out the association between lifestyle habits and illness. However, when it came to supporting behavior change interventions their views differed regarding their own responsibility and capacity.

##### Beyond screening and brief advice

The physicians who adopted a broader view on their role responsibility, beyond screening for lifestyle habits and giving brief advice on associated health risks, also saw a role for themselves in educating patients, as well as motivating and supporting them to change their behavior.

*“Primary focus should be educating patients.” U.S.*

*“Our job is to motivate them to change… to guide people*,* … if they can change*,* then we can keep them off medication …” U.S.*

*“Lifestyle issues are an important part of the work. The pedagogical role has increased.” Sweden*.

##### Setting limits on the role of promoting healthy lifestyles

The importance of defining - and limiting – their role in healthy lifestyle promotion was discussed among informants from both countries, but more extensively among the Swedes, who to a greater extent had access to other expertise and referral opportunities.

*“It is most important to address the association between lifestyle and ill-health.” Sweden*.

*“Motivational Interviewing is part of my medical role if there is no lifestyle clinic at the health center.” Sweden*.

*“The doctor’s work touches many areas*,* but the most important is the medical one. It is important to find a way of working where you limit your own role*,* and refer on to others who know better*,* such as dietitians*,* physiotherapists. It´s not my job to be a specialist in everything.” Sweden*.

#### Sharing responsibility across professional groups

Even among those informants who could see a broader role for themselves in supporting lifestyle change, they questioned whether the PCP´s were the best profession to provide healthy lifestyle counseling or whether it could be managed by others with better training. The U.S. informants pointed out that it should not only be their responsibility. They also saw opportunities for other clinical professions. In particular, nurses, dietitians and social workers were mentioned.

*“Who is the best person to counsel patients for these types of things? Is it the physician´s responsibility or is this something that could be done with nurses*,* … if they´re better people to do it*,* people who are better teachers…if there are nurses that are trained to do it*,* that would be a good thing too.” U.S.*

*“Motivational interviewing is part of the doctor’s role*,* but others can have better success due to better education.” Sweden*.

### Client receptiveness and likely impact of intervening

#### Lifestyle habits are hard to change

The impact of counseling was primarily discussed by the U.S. informants, as they were generally more involved in promoting healthy lifestyles than their Swedish counterparts. Many felt that facilitating behavior change, from several perspectives, is a difficult task that can be tiresome, but also satisfying when it leads to good outcomes. The analysis of the perceptions of client receptiveness and beliefs about their ability to make a difference, resulted in two sub-categories: confidence in the potential and doubts about impact.

#### Confidence in the potential

The hopeful ones always seemed to leave the door open for behavior change and were confident that PCPs were the best positioned to make an impact because patients trust and see them on a regular basis.

*“We*,* (PCP´s) are definitely the best people to create impact because the patients see us on a regular basis*,* and we establish a relationship with them.” U.S.*

*“If the doctors tell you to stop smoking the patient is more likely to do so.” U.S.*

#### Doubts about impact

The physicians who expressed a *pessimistic - or a realistic view* were in the majority and believed they were unlikely to succeed. These individuals felt that it’s hard for people to change; patients are not motivated and don´t follow advice, and that lifestyle is impacted by socio-economic status. Lack of success was also related to their own capacity to communicate effectively with patients; short consultation times; lack of continuity, low potential for teamwork, and few support mechanisms. Among the U.S. informants, culture was also mentioned as a barrier.

*“… Healthcare cannot affect that much. Determining factors lie outside healthcare.” Sweden*.

*“I dedicate myself to counseling but do not always think it gives so much*,* it´s not effective*,* …it needs something in between [consultations]*,* someone else*,* maybe some program. Need to work more actively between consultations.” Sweden*.

*“I tend to start people on medications the day I see them because lifestyle modification is a great idea*,* but in reality*,* people don’t do it.” U.S.*

*“…most of my patients*,* if they’re depressed*,* they want a pill*,* if they’re overweight*,* they want surgery*,*… and it’s sort of our culture and I think the onus*,* my job is to guide people to their health*,* but I can’t change their health”. U.S.*

### Self-efficacy

#### Differences in professional self-efficacy influence lifestyle counseling

The informants responses to the case vignette showed that all discussed behavioral risk factors with patients, but the number and type of risk factors covered often varied. Lifestyle change was described as a tough area and the experience and confidence needed to address lifestyle habits with patients varied among the PCP´s in both countries. All felt confident regarding tobacco use but when it came to alcohol use, physical activity and eating habits, confidence varied.

*“Smoking has become a lot easier because folks are so knowledgeable about it and there is great literature to print out for them*,* … and then there´s Chantix …” U.S.*

*“I have competence to discuss tobacco and alcohol. Diet is harder”. Sweden.*

*“Physical activity*,* there are some pretty clear guidelines on what the recommendations are*,* and I´m comfortable going over that*,* but in terms of nutrition*,* I never feel like I have enough time or quite the expertise that it takes to talk about dietary changes.” U.S.*

The U.S. informants revealed that they lacked confidence to address alcohol use because it is still a taboo subject, and there are limited resources in terms of what they can offer patients in need of support.

*“…in regard to alcohol it´s a remarkably taboo subject still. And if this guy*,* your David example [Case vignette] hadn’t had high blood pressure*,* I probably wouldn´t have asked him about his alcohol.” U.S.*

*“I definitely feel less comfortable or capable of counseling patients regarding their alcohol use and part of that is if you get into the area of you know they have a problem*,* there´s limited resources in terms of what you can do to help them*,* so I think that´s probably why we don´t address it so much…” U.S.*

Their expressed experience and confidence regarding skills in communication, motivational interviewing and counseling also varied. While some felt their experience and skills were sufficient, others believed they needed more training and practice. Sufficient skills in communication were highlighted as important in all contacts with patients. Poor communication skills contributed to the low perceived effectiveness of their risk factor management practices.

*“I have a lot of expertise in counseling*,* I think. It´s what we do all day.” U.S.*

*“I need to practice motivational interviewing.” Sweden*.

*“I have no expertise in counseling patients about lifestyle factors. I have never been trained in counseling techniques.” U.S.*

### Supporting mechanisms

#### Referral opportunities to specialist services

Although there were individual differences among the informants, the Swedish physicians appeared to have greater access to support resources such as dietitians, nurses, physiotherapists and lifestyle clinics. These specialist services and other resources reduced their perceived need to address risk factor management with patients.

*“We have access to smoking cessation*,* diabetes nurses*,* lifestyle nurses*,* asthma nurses*,* physiotherapists for exercise.” Sweden*.

U.S. physicians, on the other hand, more frequently indicated that they had limited or no access to support resources other than instruction sheets, educational materials, websites, and the New York State Quit Line for tobacco use. The majority used these resources frequently but wished for increased access to other professional groups, and other ways of working, such as team approaches, group sessions, and community interventions.

*“We have no resources*,* only written information*,* and the State Quit Line.” U.S*.

*“In the computer we have a system where I can put in the topic I want for the patient to receive instructions…” U.S.*

*“The reality so far*,* is that I pretty do it myself*,* don´t have the team yet. We don´t have a system where we have a whole team of people.” U.S*.

*“…I wish for a better system- better counseling for diet*,* exercise*,* smoking…Wish for a nurse or a social worker … the patient could come back every week or two*,* I think we could be more effective…” U.S.*

#### A system in place to routinely address behavioural risk factors

As mentioned previously, our study indicates U.S. informants were more actively engaged in promoting healthy lifestyles with patients compared to the Swedish physicians. U.S. physicians appeared to have a system in place to routinely address and document behavioral risk factors as part of existing work processes. This applied to initial appointments, as well as follow-up visits, and yearly health maintenance visits.

*“For a new patient there are standard number of things to do …. I would ask about tobacco*,* alcohol*,* seat belt*,* eye doctor*,* dentist… sex*,* diet*,* and exercise”. U.S.*

*“The nurses always ask about smoking when they bring patients in the room so that’s usually up to date. …Smoking*,* alcohol*,* recreational drug use is usually in there.” U.S.*

The U.S informants also seemed more willing to provide personalized support for lifestyle change tailored to the patient´s situation, and to be more engaged in the change process. They talked about the importance of a team-approach with the patient and saw it as a joint venture, scheduling follow-up contacts until having a “good thing” with the patient. The Swedish informants put more responsibility on the patients themselves, and utilized referral opportunities to cut down their own role and responsibility.

### Working conditions

#### Preventive ambitions constrained by working conditions

Quite a few of the informants stressed they tried to do their best based on prevailing conditions but there was an imbalance between what they wanted to achieve and what was doable. A general opinion was that neither they, nor the health service in general, do a good job for prevention and risk factor management. Many barriers were mentioned for supporting lifestyle change, especially in the U.S. Time constraints for seeing patients (BHN clinicians see a patient every 15–20 min) and also the number of patients seen in a day were primary obstacles. With a lack of time to support lifestyle change in ways they knew would be most effective, physicians felt helpless and discouraged.

*“I see patients every 20 minutes… you can´t educate people in that amount of time if they have multiple comorbidities. The health care system only wants us to triage and treat and out…” U.S.*

*“…you see all these patients and you feel sort of helpless to help them in a way that you know would be the most effective…” U.S.*

*“I know that instructing and assisting this patient on weight reduction is going to take me another 45 minutes to an hour. So I have a shortcut…” U.S*.

*“Not only the time limits in a given day*,* but also the time limits when it comes to the size of your patient handling. If you have 2000 patients*,* I’m sure you’re aware of all the… you need 90 hours a week to adequately care for them.” U.S.*

Time constraints were also identified by Swedish physicians as a barrier to lifestyle counseling. These comments indicate that an increase in the effectiveness of behavioural lifestyle interventions would require more time for patient consults.

## Discussion

Our analysis showed that in both countries, lifestyle change was seen as difficult, and physicians’ involvement was shaped by confidence, skills, time, and available support. Limited resources and constrained working conditions, particularly lack of time, were barriers contributing to doubts about the effectiveness of counseling efforts. U.S. physicians were generally more engaged in promoting healthy lifestyles and described more structured routines and greater personal responsibility, whereas Swedish physicians more often relied on referral options and shared responsibility across professional groups.

A basic and important finding was physicians´ consensus about addressing lifestyle habits as an integrated part of their role as PCP´s. This finding is a good foundation for further development of this work. Already in 2005, Brotons et al. reported that European general practitioners believed that they should provide advice on preventive and health promotion activities [6]. The challenge, which has existed for almost 20 years, is to find ways to make counseling easier and more effective in everyday practice.

Physicians´ comments also demonstrated a range and variation in perceptions that were greater among individuals vs. differences at the national level. This finding was in spite of differences such as culture, health, lifestyle and health care system.

One key difference between physicians was the tendency towards pessimism and hopefulness concerning the impact of lifestyle counselling regardless of country. A pessimistic view most likely implies less of an inclination to talk about lifestyle factors with patients, and this finding might shed light on a central explanation of deficiencies found in lifestyle counseling. Over the last decade and more, there has been a substantial increase in scientific studies demonstrating both the effect and cost-effectiveness of lifestyle counseling [3, 4]. The question is to what extent these scientific findings have translated into primary care settings. A Dutch interview study [23] identified a lack of perceived proven effectiveness of interventions as a barrier to lifestyle counseling. In a U.S. study from 2018, a belief that counseling is ineffective was significantly associated with not discussing physical activity with patients that are most at-risk [24]. In a Swedish study from 2021 on brief interventions focused on alcohol use disorders, participants expressed uncertainty about the long-term effects of the method [25]. There is an obvious and important responsibility for scientists, professional societies and healthcare authorities to spread the latest evidence concerning lifestyle counseling. We also want to emphasize that even if the scientific knowledge has increased, there is still a need for more studies, especially concerning the effects of counseling on dietary habits.

An area of possible improvement (“from pessimism to hopefulness”) is more pedagogical explanations of the effects of behavioral counseling. As also demonstrated in the interviews, physicians, in general, often judge the success of counseling based on the short-term effects they might see in behavior. As these effects usually are rather small in absolute terms, the judgements tend to be pessimistic. On the contrary, physicians´ subjective evaluations of pharmacological treatment of risk factors are often based on short-term effects on surrogate measures such as HbA1C or blood pressure. These effects are often significant even after a short period of treatment, which implies that physicians´ judgements tend to be positive towards pharmacological treatment. The challenge is to describe the large effects of behavioral change on patients´ health, implicating that rather few patients have to change behavior to achieve important effects on a group level.

We also found a clear connection between pessimism and low self-efficacy concerning lifestyle counseling. Self-efficacy is often found to be a key concept in lifestyle counseling [12, 15, 26, 27]. Thus, this finding also underlines the importance of addressing physician’s pessimism about the effects of counseling by improving their self-perceived skills.

Even if the largest differences were between individual physicians, there were also important differences between the two countries. For the U.S., PCPs unanimously requested additional resources, as they are tasked with many responsibilities. Healthy lifestyle promotion is ideally a team responsibility, where different professional competencies can support each other, as noted in other countries, such as Netherlands [23], France [28] and Sweden [16].

On the other hand, referring patients to other professions is not without complications. A finding in the initial survey [17] showing that U.S. physicians were more engaged in lifestyle counseling, might partly be explained by the fact that they had few alternatives. In the Swedish system where the responsibility for lifestyle counseling is disseminated between different professions, negative side effects may be demonstrated by this study.

From a Swedish patient perspective, the picture is somewhat multifaceted. A population survey conducted by the National Board of Health and Welfare showed that a vast majority of those who had discussed lifestyle during their latest visit to healthcare had discussed this with a physician [29]. Two thirds of the patients had the discussion with a physician alone, and another 9% had it with a physician and another professional. According to our experience, when results from this study were presented to Swedish PCPs, they were often astonished, as their understanding is that registered nurses are responsible for lifestyle counseling. This study indicates the need to clearly define the responsibility for lifestyle counseling in healthcare settings.

For Swedish family physicians, some inspiration from the U.S. might help to increase lifestyle counseling. When the U.S. physicians see a patient for the first time, a comprehensive patient history is more common, including lifestyle discussions. It also seems more common that a registered nurse prepares the patient before meeting the physician, including documenting questions about e.g. smoking in the medical record. Third, it seems much more common in the U.S. to conduct an annual analysis of the patient, including updating lifestyle data. Altogether, the U.S. PCPs in our study in general seem to work with lifestyle in a more systematic and organised way than their Swedish colleagues. Our belief is that Swedish primary care should benefit from a step towards U.S. routines in this respect.

Obviously, lifestyles are somewhat different between the two countries, especially concerning eating habits, e.g. resulting in a higher frequency of obesity in the U.S. We have discussed this question more in detail previously [18]. To summarize, there were no differences in smoking and alcohol consumption in the regions compared, and no clear differences in self-reported physical activity. Accordingly, the differences in lifestyle should explain only a minor part of the differences in attitudes and practice that we have found. One of these differences in counseling was about alcohol, which, contrary to the other lifestyle habits, was more common in Sweden. U.S. physicians regarded alcohol as somewhat of a taboo subject, and felt they lacked support and resources, which likely explains the less frequent alcohol counseling noted in the U.S. In Sweden, population-based surveys showed that alcohol counseling has increased significantly between 2010 and 2017 [30], coinciding with the release of the 2011 National Guidelines for Preventive Methods by the National Board of Health and Welfare. Furthermore, a recent study found that alcohol counseling was more common in Swedish primary care compared to the Netherlands [13]. Even if alcohol is the one of the four major lifestyle habits least discussed at visits to healthcare according to the patients [31], from an international perspective. Swedish PCP´s seem to discuss alcohol more frequently as compared to other countries.

### Strengths and limitations

Strengths of this study include the number and length interviews, and the many years of work experience represented in our sample. A majority of physicians accepted the invitation to participate. One potential reason may have been that the interviewers (LJ and JD) were well-known among PCP´s in the county of Dalarna and in BHN counties, respectively, and that both were PCP´s themselves. Unfortunately, the reporting of the study was delayed for several reasons, including illness. Over this time period, the recruitment of physicians to primary care has become increasingly difficult in both countries. In Sweden, national population-based surveys show an increase in the discussion of all four lifestyle habits in primary care since 2015 [31]; however, we are not able to confirm similar observations in U.S. studies. Aside from this observation, we are not aware of changes in the two countries that would have a major impact on the study findings. A weakness of the study is that the Swedish interviews were conducted in only two out of 21 regions, and the U.S. interviews in only one health system in one state. Thus, we may have missed other important perspectives, especially in the U.S. given the complexity of U.S. health systems. Still, we think this study generates notable hypotheses, and can serve to inspire further research about lifestyle counseling in family practice. To the best of our knowledge, this is the first qualitative study comparing countries in this respect.

## Conclusions

There was consensus in both countries about addressing lifestyle habits as an integrated part of their role as primary care physicians. The study suggests that physicians were uncertain about the effectiveness of interventions concerning unhealthy lifestyles, implying a need for further dissemination of the latest evidence for such interventions. Physicians requested a team approach for promoting healthy lifestyles, and a clearly defined role for physicians should be developed.

## Supplementary Information

Additional file 1: Case study vignette (Word file)

Additional file 2: Questions in the end of the interview - Tobacco use, alcohol use, physical activity, eating habits (Word file)

## Data Availability

The datasets used in this study are not publicly available due to the potential of compromising individual privacy. Open data sharing was not customary at the time of this study and the research participants did not give consent for this. The datasets are available from the corresponding author on reasonable request.

## Abbreviations

U.S: United States
PCP: Primary care physician
