# Supplementary material for: U.S. and Swedish primary care physicians’ views on promoting healthy lifestyles: a qualitative study
Source: BMC Prim Care. 2026 Jun 17;27:235. doi: 10.1186/s12875-026-03407-1 (PMC13273973; doi:10.1186/s12875-026-03407-1)
Supplement: Supplementary file 1 — Additional file 1: Case study vignette (Word file) [file 12875_2026_3407_MOESM1_ESM.docx]

**Case study vignette**

*Presented to the referent as a text:*

Case:

David is 47 years old, no diseases or medication, but surgery for a knee injury. He has twice used the blood pressure monitor from his sister (she is medicating for hypertension): 165/102 and 160/105. He is a little bit worried about the pressure and you meet him the day after he has called your clinic. You have never met David before. He looks healthy, but you notice abdominal obesity.

Q: Describe step by step what happens when you meet David!

If the referent asks about specific life style or social data, the interviewer reveals the facts for the particular life style habit:

Smoking: Daily, about 15 cigarettes

Smokeless tobacco: No

Alcohol: The patient says that he usually drinks a beer daily, and a little more on weekends.

If the doctor asks probing questions, the consumption is: Monday – Thursday altogether around 4 beers (0.5 l, 4.5 % (weight percent)).

Friday and Saturdays 3 – 5 beers, and about 3 glasses of hard liqeur

Physical activity: Gym for muscle training 1 – 2 times/weekly in periods, not the last four months. Otherwise physical inactive

Drugs: No

Stress: No feelings of stress

Sleep: No problems

Diet: No special diet, eats “normal”

Family: Married, 2 children have left the home, 24 och 21 years old

Social: Truck driver, working full time, likes his job, no economic problems. Completed high school/gymnasium.

Ethnicity, religion: Born in USA/Sweden, Lutheran

Explore: You have described communication about (life style habits)…..(refer to the interview)….. what would you call what you have been doing, if you use one or two or three words?

If there is a need to probe: Would you call it Advice? Counseling? Information? Conversation? Dialogue? Or something else?

If not described as counseling: How do you understand the concept of counseling? What else should you have done to call it counseling?

If described as counseling: If you did less, would you still call it counseling?

Explore the whole process from welcoming the patient until he leaves the room.

Explore: Referral?

Explore: Follow-up? By yourself? By others? If follow-up by the referent, explore the communication during that meeting.

Explore: If the referent don’t ask about any life style habits, present him with some life style data and ask about the communication after the receivement of that information.
