# Supplementary material for: U.S. and Swedish primary care physicians’ views on promoting healthy lifestyles: a qualitative study
Source: BMC Prim Care. 2026 Jun 17;27:235. doi: 10.1186/s12875-026-03407-1 (PMC13273973; doi:10.1186/s12875-026-03407-1)
Supplement: Supplementary file 2 — Additional file 2: Questions in the end of the interview - Tobacco use, alcohol use, physical activity, eating habits (Word file) [file 12875_2026_3407_MOESM2_ESM.docx]

Additional file 2

Questions in the end of the interview

Tobacco use, alcohol use, physical activity, eating habits

In general, how important do you think it is to provide advice to patients on these lifestyle habits?

To what extent are you counseling patients about these lifestyle habits in your clinical work?

To what extent do you have expertisevetions and ca 850 g
1 in counseling patients about these lifestyle habits?

As compared with your current practice, how would you like to change the extent to which you discuss these lifestyle habits with patients?

|  |  |  |  |  |
| --- | --- | --- | --- | --- |
|  |  |  |  |  |
|  |  |  |  |  |
|  |  |  |  |  |
|  |  |  |  |  |

|  |  |  |  |  |  |
| --- | --- | --- | --- | --- | --- |
|  |  |  |  |  |  |
|  |  |  |  |  |  |
|  |  |  |  |  |  |
|  |  |  |  |  |  |
